# Factors Affecting Thanatosis in the Braconid Parasitoid Wasp *Heterospilus prosopidis*

**DOI:** 10.3390/insects12010048

**Published:** 2021-01-10

**Authors:** Mio Amemiya, Kôji Sasakawa

**Affiliations:** Laboratory of Zoology, Department of Science Education, Faculty of Education, Chiba University, 1-33 Yayoi-cho, Inage-ku, Chiba-shi, Chiba 263-8522, Japan

**Keywords:** age, background color, death-feigning, sex, temperature, hymenoptera

## Abstract

**Simple Summary:**

Thanatosis is an antipredator behavior widely recognized in insects, but our knowledge of this behavior in Hymenoptera is insufficient. We examined the effects of sex, age, temperature, and background color on thanatosis in the parasitoid wasp *Heterospilus prosopidis* under laboratory conditions, and found that some of these factors have significant effects on thanatosis of this species.

**Abstract:**

Thanatosis, also called death feigning, is often an antipredator behavior. In insects, it has been reported from species of various orders, but knowledge of this behavior in Hymenoptera is insufficient. This study examined the effects of sex, age (0 or 2 days old), temperature (18 or 25 °C), and background color (white, green, or brown) on thanatosis in the braconid parasitoid wasp *Heterospilus prosopidis*. Thanatosis was more frequent in 0-d-old individuals and in females at 18 °C. The duration of thanatosis was longer in females, but this effect of sex was weaker at 18 °C and in 0-d-old individuals. The background color affected neither the frequency nor duration. These results were compared with reports for other insects and predictions based on the life history of this species, and are discussed from an ecological perspective.

## 1. Introduction

Thanatosis, also called death feigning, is often an antipredator behavior. In insects, it has been reported from species in the orders Phasmatodea, Lepidoptera, Plecoptera, Hemiptera, Orthoptera, Coleoptera, Odonata, and Hymenoptera (reviewed in [1,2]). Several coleopteran species have been established as model systems, and various factors affecting thanatosis have been identified in laboratory studies. For example, sex [1,3,4], temperature [4], age [5], body size [3], starvation [1], and behavior before thanatosis [6,7] all affect the frequency or duration of thanatosis, although the effects vary among species. Compared with reports on Coleoptera, few reports of thanatosis in species from other orders are available, and even fewer studies have focused on factors that can affect thanatosis. Studies of unexamined species are important not only for identifying the factors affecting thanatosis in those species but also for elucidating the general mechanism of thanatosis across the class Insecta.

In Hymenoptera, thanatosis has been reported in one bee species [8], several parasitoid wasps [2], several ants ([9] and references therein), and one sawfly species [10]. However, only King and Leaich [2] focused on and systematically examined factors that can affect thanatosis in a parasitoid wasp, *Nasonia vitripennis*; the others focused on other behaviors, simply noting the presence of thanatosis without examining it further. Thus, our current knowledge of thanatosis in Hymenoptera is insufficient. This study examined thanatosis in *Heterospilus prosopidis* Viereck, a species in the hymenopteran family Braconidae. This species is a solitary ectoparasitoid on the larvae and pupae of the seed beetle subfamily Bruchinae (Coleoptera, Chrysomelidae). In the field, adult *H. prosopidis* are predominantly found on seed pods drying on the branches of *Prosopis* (Mimosoideae), which are host plants of their bruchid hosts [11,12]. Several laboratory-maintained strains of this wasp species exist and have been used to study predator–prey dynamics [13,14], behavior [15], and genetics [16,17].

While rearing our *H. prosopidis* strains, we found that physical stimuli (e.g., touching with tweezers) induced thanatosis in adult wasps. Given that in the field, *H. prosopidis* adults spend most of their time on seed pods on trees, thanatosis, which is followed by dropping to the ground, is thought to serve an antipredator function. Here, the effects of sex, age, temperature, and background color on the frequency and duration of thanatosis were investigated in *H. prosopidis* under laboratory conditions. Concerning temperature and age, we hypothesized that individuals at lower temperatures and younger individuals exhibit higher frequency and longer duration of thanatosis because these individuals would show lower activity levels, where thanatosis would be more effective than other anti-predator behaviors such as escape. Although no differences in thanatosis characteristics between males and females were reported in *N. vitripennis* [2], our study of *H. prosopidis* included the sex as a possible factor. This is because although the effects vary among species, it has been reported that in some other insect species, the sex affects thanatosis through interactions with other factors. These three factors were arranged in a factorial design so that the effects of all interaction terms could be evaluated. Although the effects of these factors on thanatosis have been investigated in a variety of insects, relatively few studies have examined the interactions among the factors. Given that laboratory-based research generally reflects only a small fraction of factors in the real world, laboratory-based research incorporating more factors and their interactions is important as a link between results of laboratory-based research and actual conditions in the field.

The remaining factor, background color, assumes the situation that wasps displaying thanatosis experience in their natural habitat. The habitat of *H. prosopidis* is semi-desert grassland, and the ground surface consists of herbaceous plants and sand and gravel that vary in color from yellowish brown (some of the sand and gravel), similar to the body color of *H. prosopidis*, to green (herbaceous plants), completely different from the wasp body color. Differences in the ease of discovery due to differences in color between the ground surface and parasitoid body may influence thanatosis in *H. prosopidis*. Thus, we hypothesized that *H. prosopidis* shows thanatosis of low frequency and short duration when background colors are not similar to its own body color.

## 2. Materials and Methods

### 2.1. Insect Preparation

We used *H. prosopidis* strain AZ2007, which was established in 2007 from individuals collected in Arizona, USA, and has been maintained on *Callosobruchus maculatus* (Fabricius) infesting the cowpea *Vigna unguiculata* (Linnaeus) in our laboratory.

Wasps for the experiment were reared on cowpeas containing one late fourth-instar larva of the host beetle. The infested cowpeas were kept individually in glass Petri dishes (7-cm diameter, 1.5 cm high), which were kept in an incubator maintained at 29 °C under a L16:D8 h photoperiod until 9 days after oviposition. On day 10, they were removed from the incubator and stored on a sturdy laboratory bench in a temperature-controlled room maintained at 22 °C under a L16:D8 photoperiod. The emerged adults did not experience vibrations of the incubator that might affect the thanatosis response in later experiments. The emergence of adults was checked every 24 h. The emerged wasps were maintained without mating in a Petri dish with the cowpea containing the host remains until subsequent procedures.

### 2.2. Observations of Thanatosis

The effects of the following four factors on the frequency and duration of thanatosis were examined: sex (male or female), temperature (18 or 25 °C), age (0 or 2 days old), and background color (white, green, or brown). Temperature was regulated using an air conditioner in the laboratory. The actual temperature under the 18 °C treatment averaged 18.5 °C (range 16.5–19.6 °C), and that under the 25 °C treatment averaged 25.2 °C (range 24.3–26.1 °C). Before the experiment, the wasps were maintained at each temperature treatment for at least 15 min. Wasps 0–24 h after emergence were classified as 0 d old, and those 48–72 h as 2 d old. According to a previous study [18], the longevity of *H. prosopidis* without a food source (the same feeding treatment as in this experiment) is 7–19 (mean 15.7) days. The background color was adjusted with the paper under the Petri dish. For white, white copy paper was used. For green and brown, Toyo Origami Paper Single Color 116 Green and 150 Brown (Toyo, Tokyo, Japan) were used, respectively.

Of the four factors, sex, temperature, and age were arranged in a 2 × 2 × 2 factorial design, with white as the background color. The effect of background color was first examined under the “female, 18 °C, and 0-d-old” treatment, which showed the highest frequency of thanatosis among all of the white-background color treatments. The experiment was also performed under the “female, 25 °C, and 2-d-old” treatment, which had the lowest frequency of thanatosis in females, the same sex as in the first experiment. Subsequent experiments were conducted with the 12 treatments shown in Table 1.

A Petri dish containing a wasp and the cowpea, each with the respective experimental treatment, was placed on a sturdy laboratory bench in a lighted room. The wasp behavior was recorded at 29.97 fps from above using a video camera (HDR-PJ670; Sony, Tokyo, Japan) attached to a photo stand. The video data were analyzed using the software QuickTime for Windows v.7.75 (Apple, Cupertino, CA, USA). Thanatosis was induced by opening the Petri dish and tapping the abdomen from one side lightly with the tip of a pair of tweezers. The stimuli by tapping were given up to five times until thanatosis was induced. The duration of thanatosis was determined from the number of frames in the video. The sample size was 42–64 for each treatment.

### 2.3. Statistical Analysis

All analyses were performed using generalized linear models (GLMs) in the statistical software R v.3.4.3 [19]. For thanatosis frequency, the induction of thanatosis in each individual was considered as a binomial response variable (0, not induced; 1, induced), and GLMs with a binomial error structure and logit link function were constructed. For thanatosis duration, the number of frames was considered as a quantitative response variable, and GLMs with a Gaussian error distribution and an identity link function were constructed. In the factorial experiment examining sex, temperature, and age, the full model (i.e., one containing all the main effect terms and all interaction terms as qualitative explanatory variables) was constructed first. Then, parsimonious models were selected using the function step AIC in the R package MASS; this function selects the most parsimonious model based on minimizing the Akaike information criterion (AIC). When comparing background color effects, background color was included as a single qualitative explanatory variable. Because the background color term had three levels, Tukey’s multiple comparison was performed using the function glht in the R package multcomp. The raw data of the experiments are available in Table S1.

## 3. Results

The frequency of thanatosis varied among the treatments, ranging from 55.1% to 100%. The duration of thanatosis also varied among treatments, with mean values of 801–4408 frames, which correspond to 26.7–147.1 s. For the effects of sex, temperature, and age on the frequency of thanatosis, the parsimonious GLM selected by AIC included all the main effect terms and the sex × temperature and sex × age interaction terms. Using 2-d-old males at 25 °C as the reference group, the frequency of thanatosis was higher in 0-d-old individuals and in females at 18 °C (Table 2a). The parsimonious GLM for the duration included the same explanatory variables; with 2-d-old males at 25 °C as the reference group, it showed that the duration was longer in females, but the effect was weaker at 18 °C and in 0-d-old individuals (Table 2b). The background color had no effect on either the frequency or duration of thanatosis (Table 3).

## 4. Discussion

The frequency of thanatosis was slightly higher in females at 18 °C compared to other settings, and the duration was longer in females. Although the experimental methods have differed for each species, these results are generally consistent with the observed higher frequency of thanatosis in individuals with low activity levels reported in the sweetpotato weevil *Cylas formicarius* (Fabricius) [6], parasitoid wasp *N. vitripennis* [2], and *Callosobruchus* seed beetles [4]. In *C. formicarius*, thanatosis was more frequent in the light period, when activity levels are generally low for this nocturnal species, and in a state of rest. In *N. vitripennis*, individuals with lower activity had a higher frequency of thanatosis. In *Callosobruchus chinensis* (Linnaeus) and *C. maculatus*, thanatosis was more frequent at lower ambient temperatures (measured at 15, 20, 25, 30, and 35 °C), which cause low activity. The high thanatosis frequency in *H. prosopidis* females at 18 °C is probably associated with low activity caused by low temperatures, as in the two *Callosobruchus* species. The high frequency of thanatosis in 0-d-old *H. prosopidis* may be associated with low activity levels, such as a low frequency of flight, which may be caused by weakly sclerotized body parts.

The observation that the duration of thanatosis was longer in females than in males can be explained by the difference of mating system between males and females. In *H. prosopidis*, as in many other hymenopterans, a single copulation is sufficient for females, but multiple copulations are possible for males [16]. Under such circumstances, the decrease in mating opportunities due to thanatosis and resulting decrease in fitness are greater in males than in females. This assumption predicts that the duration of thanatosis would be shorter in males than in females, which is consistent with our results. The relationship between future mating opportunities and thanatosis duration can be examined in another way. In this study, virgin individuals were used for both sexes to control mating status, which could affect thanatosis. However, mating status can be controlled at the individual level. Therefore, the relationship between the number of mating experiences and thanatosis duration can be examined at the individual level. By performing the analysis in each sex, the relationship between future mating opportunities and thanatosis duration can be investigated, with the effects of sex removed.

Some issues remain unresolved regarding the duration of thanatosis. First, the observed effects of age differed from those of Kuriwada et al. [5], the only study examining the effects of age on the thanatosis duration in insects. They examined the thanatosis duration in the sweetpotato weevil *C. formicarius* and found that the duration was shorter in older females than in younger females. They interpreted this result as consistent with the prediction based on life-history theory that risky behaviors such as reproduction and foraging increase with age, but predator avoidance such as thanatosis decreases with age [5]. However, our results that in *H. prosopidis*, the duration of thanatosis was longer in older females than in younger females were contrary to this prediction. Note that the focal prediction based on life history theory assumes that there is a trade-off between anti-predatory behavior and behaviors such as reproduction and foraging [5]. However, females of the family Braconidae, which includes *H. prosopidis*, are monogamous and do not perform host feeding; thus, there may be no trade-off between the behaviors. If so, this may explain our results regarding the effects of age on the duration of thanatosis.

The second unresolved issue is that the effect of temperatures on thanatosis duration appears to be inconsistent with that on thanatosis frequency. The duration of thanatosis was longer in females at 25 °C than in females at 18 °C, suggesting that thanatosis is more effective at high moderate temperature. However, the frequency of thanatosis was higher in females at 18 °C than in females at 25 °C, suggesting that thanatosis is more effective at low moderate temperature. This discrepancy may be due to factors not addressed here, such as individual size or other physiological conditions. Importantly, this female-specific enigmatic effect of temperature was detected by statistical models that include interaction terms between sex and temperature. When analyses are performed separately for the male and female data, effects of temperature on either the frequency or duration cannot be detected in either sex (Tables S2 and S3). These results confirm the importance of the experimental designs, including the interaction effects between factors.

To understand the mechanism of thanatosis in a species, information on its predators and predation pressures in the field is necessary. However, the information is not available for most insect species in which thanatosis has been examined, including other parasitoid wasp [20,21]. Although birds, lizards, spiders, and other insects are generally considered to be predators of parasitoid wasps, to our knowledge, no species of parasitoid wasps has been systematically examined for predators and predation pressures in the field. Although the predators of and predation pressure on *H. prosopidis* in the field also remain to be determined, our study may provide some insights into this issue. We first hypothesized the existence of low-frequency and short-duration thanatosis in wasps based on background colors that are not similar to their body color. However, neither the frequency nor the duration differed among the background color treatments. One possible explanation for this result is that there has been no selection against wasps that remain immobile on background colors that make them conspicuous after falling from seed pods on the branches. If so, animals capable of tracking and preying on wasps after they fall to the ground (e.g., birds and flying predatory insects) may not be the primary predator of *H. prosopidis*. Future studies to identify predators in the field are needed.

A comparison of our results in *H. prosopidis* with those in *N. vitripennis* [2] revealed some similarities. For example, our finding of no difference in thanatosis frequency between males and females in *H. prosopidis* at 25 °C is virtually identical to that in *N. vitripennis*, where there was no difference in the frequency between males and females within 3 days of emergence at 26 °C. In addition, background color had no effect on the frequency of thanatosis in both species. At this stage, it is unclear whether these common features have the same ecological significance. Future studies of thanatosis in more hymenopteran species will give insights into this issue.

## 5. Conclusions

The present study revealed that the frequency and duration of thanatosis are affected by sex, age, and temperature, but not by background color in *H. prosopidis*. Some of these results are compatible with previous reports on other insect species and predictions based on the life history of this species.

## Figures and Tables

**Table 1:** Frequency and duration of thanatosis in *Heterospilus prosopidis* under various treatments.

Factors				% Thanatosis Response (*n*)	Duration ^1^ of Thanatosis (*n*)
Sex	Temperature (°C)	Age (days)	Background Color		
Male	18	0	White	83.0 (47)	1093 ± 143 (39)
Male	18	2	White	55.1 (49)	1129 ± 471 (27)
Male	25	0	White	83.0 (47)	801 ± 199 (39)
Male	25	2	White	62.0 (50)	833 ± 176 (31)
Female	18	0	White	89.1 (46)	1546 ± 218 (41)
Female	18	2	White	77.8 (45)	2934 ± 823 (35)
Female	25	0	White	72.9 (48)	2609 ± 514 (35)
Female	25	2	White	66.7 (42)	4408 ± 1266 (28)
Female	18	0	Green	100 (50)	1484 ± 179 (50)
Female	18	0	Brown	92.2 (64)	1323 ± 231 (59)
Female	25	2	Green	74.5 (47)	1971 ± 476 (35)
Female	25	2	Brown	61.7 (47)	2493 ± 948 (29)

^1^ Units are the number of frames, in a video recorded at 29.97 fps.

**Table 2:** Parsimonious generalized linear models selected by AIC for the effects of sex, temperature, and age on the frequency and duration of thanatosis in *H. prosopidis*. GLM indicates a reference group as an intercept. Here, the reference group is 2-d-old males at 25 °C in models of both frequency and duration.

	β (Mean ± SE)	*z*-Value	*p*
(a) Model for effects on frequency			
Intercept	0.44 ± 0.26	1.670	0.095
Female	0.15 ± 0.39	0.382	0.702
18 °C	−0.18 ± 0.33	−0.559	0.576
0-d old	1.24 ± 0.34	3.623	<0.001
Female × 18 °C	0.98 ± 0.49	1.995	0.046
Female × 0-d old	−0.73 ± 0.5	−1.466	0.143
(b) Model for effects on duration			
Intercept	834 ± 485	1.718	0.087
Female	3449 ± 695	4.961	<0.001
18 °C	293 ± 542	0.541	0.589
0-d old	−34 ± 548	−0.061	0.951
Female × 18 °C	−1542 ± 764	−2.019	0.045
Female × 0-d old	−1540 ± 768	−2.006	0.046

**Table 3:** Pairwise comparisons of generalized linear models of the effects of background color on the frequency and duration of thanatosis in *H. prosopidis* females.

	β (Mean ± SE)	*z*-Value	*p*
(a) Effects on the frequency at low temperature and younger age			
Brown vs. white	0.36 ± 0.66	0.548	0.827
Green vs. white	17.46 ± 1520.85	0.011	1.000
Green vs. brown	17.1 ± 1520.85	0.011	1.000
(b) Effects on the frequency at high temperature and older age			
Brown vs. white	−0.22 ± 0.44	−0.487	0.877
Green vs. white	0.38 ± 0.47	0.806	0.699
Green vs. brown	0.59 ± 0.45	1.321	0.383
(c) Effects on the duration at low temperature and younger age			
Brown vs. white	−223 ± 309	−0.721	0.751
Green vs. white	−62 ± 320	−0.193	0.980
Green vs. brown	161 ± 292	0.551	0.846
(d) Effects on the duration at high temperature and older age			
Brown vs. white	−1916 ± 1320	−1.451	0.315
Green vs. white	−2437 ± 1263	−1.929	0.130
Green vs. brown	−522 ± 1251	−0.417	0.909

## Data Availability

The data of this study are available in Table S3 of Supplemental Materials.

## Supplementary Materials

The following are available online at https://www.mdpi.com/2075-4450/12/1/48/s1, Table S1: Raw data of the experiments. Table S2: Generalized linear models for the effects of temperature and age on the frequency and duration of thanatosis in male *H. prosopidis*, Table S3: Generalized linear models for the effects of temperature and age on the frequency and duration of thanatosis in female *H. prosopidis*.
