# Supplementary material for: Factors Affecting Thanatosis in the Braconid Parasitoid Wasp Heterospilus prosopidis
Source: Insects. 2021 Jan 10;12(1):48. doi: 10.3390/insects12010048 (PMC7826778; doi:10.3390/insects12010048)
Supplement: Supplementary file 1 [file insects-12-00048-s001.zip › insects-1062417-TablesS2&S3-xml.pdf]

Table S2. Generalized linear models for the effects of temperature and age on the frequency and duration of thanatosis in male *H. prosopidis*. The reference group (intercept) is 2-d-old individuals at 25°C in models of both frequency and duration.

	β (mean ± SE)	*z*-value	*p*
(a) Model for effects on frequency			
Intercept	0.49 ± 0.29	1.680	0.093
18°C	–0.28 ± 0.41	–0.696	0.486
0-d old	1.09 ± 0.49	2.255	0.024
18°C × 0-d old	0.28 ± 0.68	0.416	0.678
(b) Model for effects on duration			
Intercept	833 ± 259	3.219	0.002
18°C	296 ± 379	0.780	0.437
0-d old	–32 ± 347	–0.092	0.927
18°C × 0-d old	–4 ± 500	–0.008	0.994

Table S3. Generalized linear models for the effects of temperature and age on the frequency and duration of thanatosis in female *H. prosopidis*. The reference group (intercept) is 2-d-old individuals at 25°C in models of both frequency and duration.

	β (mean ± SE)	*z*-value	*p*
(a) Model for effects on frequency			
Intercept	0.69 ± 0.33	2.118	0.034
18°C	0.56 ± 0.49	1.153	0.249
0-d old	0.30 ± 0.46	0.645	0.519
18°C × 0-d old	0.55 ± 0.75	0.737	0.461
(b) Model for effects on duration			
Intercept	4408 ± 798	5.523	<0.001
18°C	–1474 ± 1071	–1.377	0.171
0-d old	–1800 ± 1071	–1.681	0.095
18°C × 0-d old	–411 ± 1466	0.284	0.777
